# A Combinatorial Approach to Optimize the Production of Curcuminoids From Tyrosine in *Escherichia coli*

**DOI:** 10.3389/fbioe.2020.00059

**Published:** 2020-02-07

**Authors:** Joana L. Rodrigues, Daniela Gomes, Lígia R. Rodrigues

**Affiliations:** Centre of Biological Engineering, University of Minho, Braga, Portugal

**Keywords:** curcuminoids, biosynthesis, *E. coli*, caffeic acid O-methyltransferase, co-culture engineering, biosynthetic pathway

## Abstract

Curcuminoids are well-known for their therapeutic properties. However, their extraction from natural sources is environmentally unfriendly, expensive and limited by seasonal variability, highlighting the need for alternative production processes. We propose an optimized artificial biosynthetic pathway to produce curcuminoids, including curcumin, in *Escherichia coli*. This pathway involves six enzymes, tyrosine ammonia lyase (TAL), 4-coumarate 3-hydroxylase (C3H), caffeic acid O-methyltransferase (COMT), 4-coumarate-CoA ligase (4CL), diketide-CoA synthase (DCS), and curcumin synthase (CURS1). Curcuminoids pathway was divided in two modules, the first module included TAL, C3H and COMT and the second one 4CL, DCS and CURS1. Optimizing the first module of the pathway, from tyrosine to ferulic acid, enabled obtaining the highest ferulic acid titer reported so far (1325.1 μM). Afterward, ferulic acid was used as substrate to optimize the second module of the pathway. We achieved the highest concentration of curcumin ever reported (1529.5 μM), corresponding to a 59.4% increase. Subsequently, curcumin and other curcuminoids were produced from tyrosine (using the whole pathway) in mono-culture. The production increased comparing to a previously reported pathway that used a caffeoyl-CoA O-methyltransferase enzyme (to convert caffeoyl-CoA to feruloyl-CoA) instead of COMT (to convert caffeic to ferulic acid). Additionally, the potential of a co-culture approach was evaluated to further improve curcuminoids production by reducing cells metabolic burden. We used one *E. coli* strain able to convert tyrosine to ferulic acid and another able to convert the hydroxycinnamic acids produced by the first one to curcuminoids. The co-culture strategies tested led to 6.6 times increase of total curcuminoids (125.8 μM) when compared to the mono-culture system. The curcuminoids production achieved in this study corresponds to a 6817% improvement. In addition, by using an inoculation ratio of 2:1, although total curcuminoids production decreased, curcumin production was enhanced and reached 43.2 μM, corresponding to an improvement of 160% comparing to mono-culture system. To our knowledge, these values correspond to the highest titers of curcuminoids obtained to date. These results demonstrate the enormous potential of modular co-culture engineering to produce curcumin, and other curcuminoids, from tyrosine.

## Introduction

**FIGURE 1:**
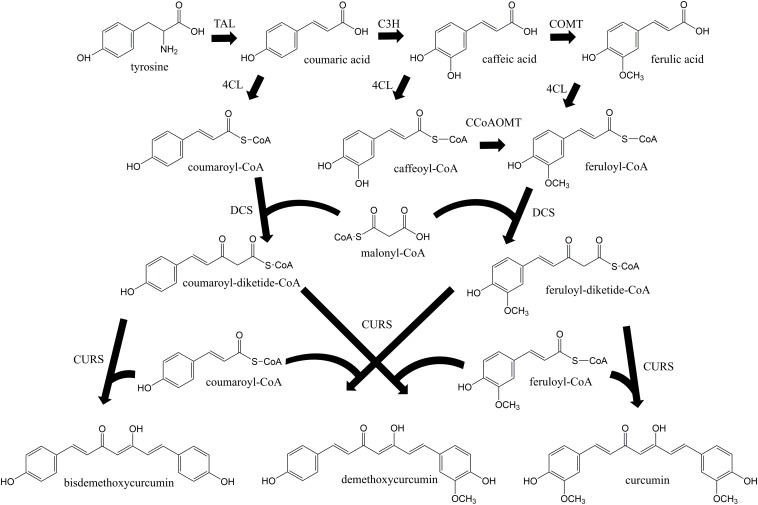
Curcuminoids biosynthetic pathway constructed in *E. coli*. Two molecules of CoA esters (coumaroyl-CoA or/and feruloyl-CoA) and one molecule of malonyl-CoA are needed to produce one molecule of curcuminoid (bisdemethoxycurcumin, demethoxycurcumin or curcumin). TAL, tyrosine ammonia lyase; C3H, 4-coumarate 3-hydroxylase; COMT, caffeic acid O-methyltransferase; CCoAOMT, caffeoyl-CoA O-methyltransferase; 4CL, 4-coumarate-CoA ligase; DCS, diketide-CoA synthase; CURS, curcumin synthase. Adapted from Rodrigues et al. (2015b).

Curcuminoids are natural phenylpropanoids extracted from the plant *Curcuma longa* that have innumerous therapeutic properties including antioxidant (Wang et al., 2019), anti-inflammatory (Dai et al., 2018), anticancer (Murray-Stewart et al., 2018; Ravindranathan et al., 2018), and cholesterol-lowering (Ferguson et al., 2018). They have also been reported as protective agents for several neurodegenerative diseases (Maiti and Dunbar, 2018). Curcumin, the curcuminoid that exhibits the highest biological activity, holds an estimated market size of USD 133 million by 2025 (Value Market Research, 2019). Different curcuminoids have different therapeutic properties, therefore being important to assess their individual bioactivity as pure compounds. However, they have been mainly isolated from turmeric in low amounts as a mixture of curcumin, demethoxycurcumin, bisdemethoxycurcumin, and other curcuminoids. Pure curcuminoids are very expensive and rare. Besides low purity, the plant extraction also presents other disadvantages such as long periods of growth and seasonal variations. Thus, cost-effective and environmentally friendly methods to obtain pure curcuminoids ought to be developed. In the past decade, several efforts have been made to produce curcuminoids using synthetic biology and metabolic engineering approaches (Katsuyama et al., 2008, 2010; Wang et al., 2013, 2015; Rodrigues et al., 2015b, c, 2017; Couto et al., 2017; Kim et al., 2017; Fang et al., 2018; Kang et al., 2018; Kan et al., 2019). Curcuminoids have been produced intracellularly in *Escherichia coli* host according to the biosynthetic pathway in Figure 1. The curcumin biosynthetic pathway is complex requiring the overexpression of six genes from plant, bacterial and fungal sources. First, tyrosine is converted to coumaric acid by tyrosine ammonia lyase (TAL). Afterward, caffeic acid is produced from coumaric acid by 4-coumarate 3-hydroxylase (C3H). Then, two different approaches can be used. The enzyme 4-coumarate-CoA ligase (4CL) can convert caffeic acid to caffeoyl-CoA, and caffeoyl-CoA O-methyl transferase (CCoAOMT) (Rodrigues et al., 2015b) converts caffeoyl-CoA to feruloyl-CoA. In the other approach, caffeic acid is converted to ferulic acid by caffeic acid O-methyltransferase (COMT) and then to feruloyl-CoA by 4CL (Wang et al., 2015; Kang et al., 2018). Next, two molecules of feruloyl-CoA and one of malonyl-CoA are converted to curcumin by diketide-CoA synthase (DCS) and curcumin synthase (CURS1) from *C. longa* or by curcuminoid synthase (CUS) from *Oryza sativa*. Since 4CL, DCS, CURS1, and CUS present broad substrate specificity (Rodrigues et al., 2015b, c), other curcuminoids are also produced. The highest curcumin concentration reported previously using ferulic acid as substrate was 959.3 μM (353.4 mg/L) at a yield of 95.9% (Couto et al., 2017). However, curcumin production from tyrosine only reached up to 0.6 mg/L (Wang et al., 2015). Recently, the curcumin biosynthetic pathway was inserted in the *E. coli* genome and 3.8 mg/L of curcumin were produced from glucose using 4CL and DCS enzymes with mutations that confer lower translation efficiencies (Kang et al., 2018). Despite this improvement, the curcumin/curcuminoids biosynthetic pathway still needs to be optimized to further increase the titers.

In this study, we did a step-by-step optimization of the curcumin biosynthetic pathway. We started by dividing the pathway in two modules. The first one contained the genes of the pathway that converted tyrosine to ferulic acid. The second module contained the genes that converted the hydroxycinnamic acids to curcuminoids. The modules were optimized separately. The highest ferulic acid production achieved was 1325.1 μM (257.3 mg/L) and corresponded to the highest production obtained to date. The second module of the pathway was optimized by feeding ferulic acid to produce curcumin. The curcumin concentration was improved to 1529.5 μM (563.4 mg/L), the highest production achieved until now. When the complete pathway was assembled in one strain (monoculture system), the total curcuminoids (19.0 μM; 6.87 mg/L) and curcumin (16.6 μM; 6.1 mg/L) production from tyrosine also increased compared to the production obtained in previous studies (Rodrigues et al., 2015b; Wang et al., 2015). However, it was still very low, mainly due to the metabolic burden caused by the overexpression of the six genes of the biosynthetic pathway. Recently, the use of co-culture engineering, where two strains carry different modules of the biosynthetic pathways, proved to improve the production of several natural compounds (Jones et al., 2017; Li et al., 2019), including curcuminoids (Fang et al., 2018). Therefore, a co-culture system that allow a cascade conversion of tyrosine to curcuminoids was tested, where an *E. coli* strain carrying the first module of the pathway (TAL, C3H, and COMT genes) produced hydroxycinnamic acids that another *E. coli* strain carrying the other three genes of the pathway (4CL, DCS, and CURS1 genes) was able to convert to curcuminoids. In the co-culture system, the production of total curcuminoids from tyrosine was 125.8 μM (41.5 mg/L). When a ratio of 2:1 (first module of the pathway: second module of the pathway) was used in the co-culture experiment, the curcumin production reached 43.2 μM (15.9 mg/L), i.e., the highest concentration obtained so far. Regarding the mono-culture system, the production of total curcuminoids and curcumin increased 6.6 and 2.6 times, respectively.

## Materials and Methods

### Strains, Plasmids, and Chemicals

*E. coli* NZY5α competent cells (NZYTech, Lisbon, Portugal) were used for molecular cloning and vector propagation and *E. coli* BL21 (DE3) (NZYTech) was used as host for expression of the biosynthetic pathway. Table 1 summarizes the characteristics of all strains and plasmids used. Synthesis and amplification of TAL, C3H, 4CL1, DCS, and CURS1 genes, as well as plasmid construction with these genes was previously described (Rodrigues et al., 2015a, b). pAC-4CL1 plasmid was kindly provided by Claudia Schmidt-Dannert (Watts et al., 2006) (Addgene plasmid # 35947). The plasmids pCas9Cr4 (Addgene plasmid #62655) and pKDsgRNA-p15A (Addgene plasmid #62656) were kindly provided by Kristala Prather (Reisch and Prather, 2015).

**TABLE 1:** Bacterial strains and plasmids used in this study.

Strains	Relevant genotype	Source
*E. coli* NZY5α	*fhuA2*Δ(*argF^–^lacZ*)U169 *phoA glnV44*Φ80 Δ(*lacZ*)M15 *gyrA96 recA1 relA1 endA1 thi-1 hsdR17*	NZYTech (MB00401)
*E. coli* BL21(DE3)	F^–^ *omp*T *gal dcm lon hsd*SB(rB- mB-) λ(DE3 ^∗^*lacI lacUV5-T7 gene 1 ind1 sam7 nin5*])	NZYTech (MB006)
*E. coli* BL21(DE3) Δ*lacZ*	F^–^ *omp*T *gal dcm lon hsd*SB(rB- mB-) λ(DE3 ^∗^*lacI lacUV5-T7 gene 1 ind1 sam7 nin5*]) Δ*lacZ*	This study

**Plasmids**	**Construct**	**Source**

pCas9Cr4	*p15A ori*, Cas9 from *Streptococcus pyogenes* under control of P*_*TET*_*, constitutive tetR, Cm^R^	Addgene (62655)
pKDsgRNA-p15A	*SC101 ori*, sgRNA targeting *p15A ori* under control of P*_*TET*_*,λred under control of P*_*araB*_*, *araC*, Spec^R^	Addgene (62656)
pKDsgRNA-LacZ	pKDsgRNA-p15A with sgRNA targeting *lacZ* instead of *p15A ori*	This study
pRSFduet-1	RSF1030 *ori*, *lacI*, double P_T__7_*_*lac*_*, Kan^R^	Novagen
pACYCduet-1	*p15A ori*, *lacI*, double P_T__7_*_*lac*_*, Cm^R^	Novagen
pUC57_COMT	pMB1 *ori*, Amp^R^; pUC57 carrying codon-optimized COMT from *Arabidopsis thaliana*	NZYTech
pRSFduet_TAL	pRSFduet-1 carrying codon-optimized TAL from *Rhodotorula glutinis*	Rodrigues et al., 2015a
pCDFduet_TAL	CloDF13 *ori*, *lacI*, double P_T__7_*_*lac*_*, Strep^R^; pCDFduet-1 carrying TAL	Rodrigues et al., 2015a
pETduet_TAL	ColE1(pBR322) *ori*, *lacI*, double P_T__7_*_*lac*_*, Amp^R^; pETduet-1 carrying TAL	Rodrigues et al., 2015a
pETduet_TAL_C3H	pETduet_TAL carrying codon-optimized C3H from *Saccharothrix espanaensis*	Rodrigues et al., 2015a
pRSFduet_C3H	pRSFduet-1 carrying C3H	Rodrigues et al., 2015a
pCDFduet_C3H	CloDF13 *ori*, *lacI*, double P_T__7_*_*lac*_*, Strep^R^; pCDFduet-1 carrying C3H	Rodrigues et al., 2015a
pCDFduet_DCS	CloDF13 *ori*, *lacI*, double P_T__7_*_*lac*_*, Strep^R^; pCDFduet-1 carrying codon-optimized DCS from *Curcuma longa*	Rodrigues et al., 2015b
pRSFduet_CURS1	pRSFduet-1 carrying codon-optimized CURS1 from *C. longa*	Rodrigues et al., 2015b
pRSFduet_CURS1_CCoAOMT	pRSFduet_CURS1 carrying codon-optimized CCoAOMT from *Medicago sativa*	Rodrigues et al., 2015b
pAC-4CL1	*p15A ori*, P*_*lac*_*, Cm^R^, pACYC184-derived plasmid carrying 4CL1 from *A. thaliana*	Addgene (35947)
pACYCduet_COMT	pACYCDuet-1 carrying COMT	This study
pRSFduet_COMT	pRSFduet-1 carrying COMT	This study
pAC_COMT	pAC-4CL1 without 4CL1 and carrying COMT	This study
pACYCduet_4CL1	pACYCduet-1 carrying 4CL1	This study
pRSFduet_CURS1_COMT	pRSFduet_CURS1 carrying COMT	This study
pCDFduet_DCS_C3H	pCDFduet_DCS carrying C3H	This study
pACYCduet_4CL1_TAL	pACYCduet_4CL1 carrying TAL	This study
pRSFduet_COMT_C3H	pRSFduet_COMT carrying C3H	This study
pRSFduet_CURS1_4CL1	pRSFduet_CURS1 carrying COMT	This study
pRSFduet_CURS1_DCS	pRSFduet_CURS1 carrying DCS	This study
pRSFduet_TAL_C3H	pRSFduet_TAL carrying C3H	This study

Tyrosine, coumaric and caffeic acid were purchased from Sigma-Aldrich (Steinheim, Germany), ferulic acid from Acros (Geel, Belgium), curcumin from Thermo Fisher Scientific (Loughborough, United Kingdom), and bisdemethoxycurcumin and demethoxycurcumin from Abcam (Cambridge, United Kingdom). Isopropyl β-D-1-thiogalactopyranoside (IPTG), super optimal broth with catabolite repression (SOC), 5-bromo-4-chloro-3-indolyl-β-D-galactopyranoside (X-Gal), and lysogeny broth (LB) medium were purchased from NZYTech. Glucose (Acros), Na_2_HPO_4_ (Scharlau, Sentmenat, Spain), MgSO_4_, KH_2_PO_4_ (Riel-deHaën, Seelze, Germany), methionine, NH_4_Cl, NaCl, CaCO_3_ (Panreac, Barcelona, Spain) and thiamine (Thermo Fisher Scientific, Loughborough, United Kingdom) were used to prepare the M9 modified salt medium. The following mineral traces and vitamins were supplemented to M9 Medium: FeCl_3_, ZnCl_2_, CoCl_2_, CuCl_2_, nicotinic acid (Riedel-deHaën), NaMoO_4_, H_2_BO_3_, pyridoxine, biotin, folic acid (Merck), riboflavin, and pantothenic acid (Sigma Aldrich). Ampicillin (AppliChem, Darmstadt, Germany), chloramphenicol, kanamycin (NZYTech), spectinomycin (Panreac), anhydrotetracycline (aTc), and arabinose (Acros) were used whenever necessary.

### Construction of Plasmids Carrying the Biosynthetic Pathway

The 4CL gene was removed from pAC plasmid using *Xba*I and *Not*I restriction enzymes. COMT gene from *Arabidopsis thaliana* (GenBank accession numbers AY062837.1) was codon-optimized and synthesized by NZYTech. The DNA sequence of the codon-optimized gene is provided in Supplementary Table S1. Afterward, COMT gene was cloned in pAC, pACYCduet-1 and pRSFduet-1. 4CL gene was amplified from pAC_4CL and cloned in pACYCduet-1. Later, for monoculture and co-culture experiments, other plasmids were also constructed. COMT gene was amplified by PCR and cloned in pRSFduet_CURS1 and pRSFduet_C3H, 4CL gene in pRSFduet_CURS1, C3H gene in pRSFduet_TAL and pCDFduet_DCS, TAL gene in pACYCduet_4CL and DCS gene in pRSFduet_CURS1. All the plasmids used in this study are presented in Table 1 and the primers (Metabion, Steinkirchen, Germany) in Supplementary Table S2.

Plasmid DNA was isolated using NucleoSpin^®^ Plasmid Miniprep Kit (Macherey-Nagel, Düren, Germany). The genes were amplified by PCR using Phusion High Fidelity DNA Polymerase (Thermo Fisher Scientific, Wilmington, DE, United States). DNA fragments were purified from agarose using NucleoSpin^®^ Gel and PCR Clean-up Kit (Macherey-Nagel). Plasmid DNA and genes were quantified in a NanoDrop instrument (ND-1000, Thermo Fisher Scientific) and were digested with the appropriate restriction endonucleases (Supplementary Table S2) (Thermo Fisher Scientific) for 3 h and purified using NucleoSpin^®^ Gel and PCR Clean-up Kit. Ligation was performed at room temperature for 1 h with T4 DNA ligase (Thermo Fisher Scientific). Chemical transformation (heat shock method) was carried out using *E. coli* NZY5α competent cells. All constructed plasmids herein described were verified by colony PCR and digestion and further confirmed by sequencing (GATC Biotech, Konstanz, Germany). After confirmation, *E. coli* BL21 (DE3) competent cells were transformed with the constructed plasmids. All the kits and enzymes were used according to the instructions provided by the manufacturers.

### Protein Analysis

*E. coli* BL21 (DE3) cells harboring pRSFduet-1, pRSFduet_ COMT, pACYCduet_COMT, and pAC_COMT were grown in LB at 37°C to an OD_600_ of 0.9. IPTG was added (at a final concentration of 1 mM) to induce protein expression, and the culture was incubated for 6 h. Samples (10 mL culture medium) were taken at time 0 and 6 h of induction. Samples were centrifuged and the cells were resuspended in Tris–HCl buffer (1 mM, pH 7.8) and were further mechanically disrupted using 1 mm glass beads (Sigma-Aldrich) and FastPrep-24 (MP Biomedicals, Salon, OH, United States) (three cycles at speed 6 m/s for 1 min, 5 min cooling on ice after each cycle). After centrifugation, the protein concentration of soluble and insoluble fractions was determined using Pierce Coomassie (Bradford) Protein Assay Kit according to the manufacturers’ instructions. The expression levels of COMT were evaluated through sodium dodecyl sulfate polyacrylamide gel electrophoresis (SDS–PAGE) (4% stacking gel and 10% running gel). Samples containing soluble or insoluble protein fraction were mixed with 2× sample buffer (65.8 mM Tris–HCl pH 6.8, 2.1% SDS, 26.3% glycerol, 0.01% bromophenol blue) and β-mercaptoethanol and were denatured in a heating block at 100°C for 5 min. The protein marker used Color Protein Standard – Broad Range (NEB). After electrophoresis, the gel was stained using Coomassie Blue R-250 for 15 min and de-stained using distilled water until a clear background was achieved.

### Construction of a *lacZ* Disrupted *E. coli* Strain

The disruption of *lacZ* from *E. coli* BL21 (DE3) was based on methods previously described using clustered regulatory interspaced short palindromic repeats – associated caspase 9 endonuclease (CRISPR-Cas9) (Reisch and Prather, 2015; Chung et al., 2017). Plasmid pKDsgRNA-*lacZ* includes a protospacer described by Chung et al. (2017) and was constructed using circular polymerase extension cloning (Reisch and Prather, 2015). The primers used are presented in Supplementary Table S3. The donor DNA (single strand) (Supplementary Table S4) was synthesized by Metabion and corresponds to two homology arms of 50 nt complementary to the *E. coli* BL21 (DE3) genome. The homology arms were homologous to the upstream region (−53 to −3 bp) and to the intergenic region of *lacZ* (1516–1566 bp) as in Chung et al. (2017).

The plasmids pCas9Cr4 and pKDsgRNA-*lacZ* were sequentially transformed in *E. coli* BL21 (DE3). The resultant strain was cultured in LB medium (10 g/L tryptone, 5 g/L yeast extract, 10 g/L NaCl) containing 25 μg/mL chloramphenicol and 50 μg/mL spectinomycin at 30°C (200 rpm). When optical density at 600 nm (OD_600_) was between 0.35 and 0.5, arabinose (1.2% (w/v) final concentration) was added to the medium to induce the λ-Red recombinase and the culture was incubated at 30°C for more 20 min. Afterward, electrocompetent cells were prepared and 10 μM donor DNA was transformed by electroporation. The cells were plated in LB agar containing chloramphenicol, spectinomycin, aTc (100 ng/mL), X-Gal (0.1 mg/mL) and IPTG (1 mM) and grew overnight at 30°C. The white colonies containing the disruption were selected, and the deletion was confirmed by colony PCR (Supplementary Table S5). The selected mutant strain was cultured at 37°C to cure pKDsgRNA-*lacZ* that has a temperature-sensitive replication origin. Then, pKDsgRNA-p15A was transformed into *E. coli* BL21 (DE3) Δ*lacZ* containing pCas9Cr4. After transformation, the cells were recovered in SOC (20 g/L tryptone, 5 g/L yeast extract, 3.6 g/L glucose, 0.186 g/L KCl, 0.5 g/L NaCl, 0.96 g/L MgCl_2_) containing aTc (100 ng/mL) for 2 h and plated in LB containing spectinomycin and aTc at 30°C overnight to induce the curing of pCas9Cr4. After confirming that the plasmid was cured, the cells were cultured at 37°C in order to cure pKDsgRNA-p15A. Finally, the mutant strain (*E. coli* BL21 [DE3] Δ*lacZ*) was transformed with the two plasmids containing the second module of the biosynthetic pathway (pCDFduet_DCS + pRSFduet_CURS1_4CL or pACYCduet_4CL + pRSFduet_CURS1_DCS) and used in co-culture systems.

### Ferulic Acid and Curcuminoids Production

*E. coli* cells for plasmid propagation and inoculum preparation were grown in LB medium at 37°C and under shaking conditions (200 rpm).

For hydroxycinnamic acids and curcuminoids production, cultures were grown at 37°C in 50 mL LB from an OD_600_ of 0.1 (≈2% inoculum volume) up to 0.9. In the co-culture experiments, the different strains (with different modules of the pathway) were separately cultivated in LB medium and these overnight cultures were then used to inoculate the co-culture experiment in LB with different inoculation ratios (≈2% inoculum volume for all the tested ratios – 1:1, 2:1, 1:2). All the experiments (mono-culture and co-culture) were performed with *E. coli* BL21 (DE3) excluding the co-cultures system experiments where each strain carried two instead of three plasmids. In these specific experiments *E. coli* BL21 (DE3) carried the first module of the pathway and *E. coli* BL21 (DE3) Δ*lacZ* carried the second one.

After reaching the target OD_600_, the protein expression was induced with IPTG (0.1 mM) and the culture was then incubated for 5 h at 26°C. Next, the cells were harvested by centrifugation, suspended, and incubated at 26°C for 63 h in 50 mL M9 medium. Tyrosine (3 mM), caffeic acid (1 mM) or ferulic acid (2 mM) and IPTG (0.1 mM) were added at time 0 of induction in modified M9 medium. The experiments occurred during 63 h after adding the substrate. In specified experiments ferulic acid (1 mM) was also added at 15 h.

Modified M9 minimal salt medium contained (per liter): 40 g glucose, 6 g Na_2_HPO_4_, 3 g KH_2_PO_4_, 1 g NH_4_Cl, 0.5 g NaCl, 15 mg CaCl_2_, 110 mg MgSO_4_, 340 mg thiamine, 149.2 mg methionine, and 5 g CaCO_3_. Trace elements (54 mg FeCl_3_, 4 mg ZnCl_2_, 4 mg CoCl_2_, 4 mg NaMoO_4_, 2 mg CuCl_2_, and 1 mg H_2_BO_3_) and vitamins (0.84 mg riboflavin, 0.084 mg folic acid, 12.2 mg nicotinic acid, 2.8 mg pyridoxine, 0.12 mg biotin, and 10.8 mg pantothenic acid) were supplemented to the medium. Depending on the plasmid(s) present in the strains, 100 μg/mL of ampicillin, 100 μg/mL of spectinomycin, 30 μg/mL of chloramphenicol and/or 50 μg/mL of kanamycin were used.

All the experiments were conducted in triplicate. Supernatant samples (1 mL) were collected for the analysis of coumaric, caffeic, and ferulic acids, while for curcuminoids 200 μL to 2 mL of culture broth with cells (whole broth) were collected.

### Estimation of the Strain-to-Strain Ratio in the Co-culture Systems

The strain-to-strain ratio in co-culture systems, where each strain carried two plasmids, was analyzed using the blue-white screening method and a method that relies on the production of curcuminoids. Strain *E. coli* BL21 (DE3) contained the first module of the pathway and *E. coli* BL21 (DE3) Δ*lacZ* contained the second module that produced curcuminoids from hydroxycinnamic acids. During the co-culture fermentation, 10 μL samples were taken and diluted 50 to 100-fold. Afterward, 10 μL of the dilution were spread in LB agar plates containing the required antibiotics, and 0.1 mg/mL X-Gal and 1 mM IPTG (for the blue-white screening method) or 0.5 mM ferulic acid and 1 mM IPTG (for curcuminoids production method). *E. coli* with disrupted *lacZ* only formed white colonies in the presence of X-gal while the other one formed blue colonies. In the other method, *E. coli* containing the second module of the pathway, in the presence of ferulic acid produced curcumin and generated yellow/orange colonies while the strain that contained the first module formed white colonies. The number of colonies of each color for each method were counted to estimate the strain-to-strain ratio in the co-culture system. It should be noted that these methods only allow to determine the ratio of viable cells.

### Curcuminoids Extraction

Curcuminoids are mostly present inside *E. coli* cells. However, when their production is very high, they can also be found in the supernatant. Therefore, whole broth was mixed with an equal volume of ethyl acetate (Thermo Fisher Scientific). The mixture was vortexed and centrifuged (2 min, 15,000 × *g*). The aqueous phase was transferred to a new tube and this process was repeated until the pellet was not colored due to curcuminoids presence. Finally, the extracts were concentrated by solvent evaporation in a fume hood, suspended with at least 200 μL of acetonitrile (Thermo Fisher Scientific) and subjected to product analysis by ultra-high-performance liquid chromatography (UHPLC).

### UHPLC Analysis

Ultra-high-performance liquid chromatography analysis was used to quantify hydroxycinnamic acids (coumaric acid, caffeic acid, and ferulic acid) and curcuminoids (curcumin, bisdemethoxycurcumin, demethoxycurcumin) using the Shimadzu Nexera-X2 system (Shimadzu Corporation, Kyoto, Japan) (CBM-20A system controller, LC-30AD pump unit, DGU-20A 5R degasser unit, SPD-M20A detector unit, SIL-30AC autosampler unit, CTO-20AC column oven) and a Kinetex^®^ 2.6 μm Polar C18 100 Å LC column (150 × 4.6 mm) (Phenomenex, Alcobendas, Spain). Mobile phase A was composed of 0.1% (v/v) of trifluoroacetic acid (Fluka) in water and mobile phase B was composed of acetonitrile. For hydroxycinnamic acids quantification, the following gradient was used at a flow rate of 1 mL/min: 10–40% mobile phase B for 16 min, 40–70% for 1 min, 70–10% for 4 min, and 10% mobile phase B for an additional 4 min. Quantification was based on the peak areas at 310 nm for caffeic acid, coumaric acid and ferulic acid and the retention time was 6.4, 8.5, and 9.2 min, respectively. For curcuminoids quantification, a gradient of 40–43% mobile phase B for 15 min and 43–70% for 3 min, 70–40% for 4 min and 40% mobile phase B for an additional 5 min was used. Curcuminoids were detected at 425 nm of absorbance and the retention time was 12.1 min for bisdemethoxycurcumin, 13.2 min for demethoxycurcumin, and 14.3 min for curcumin.

## Results and Discussion

### Rational Design and Production of Ferulic Acid From Caffeic Acid Using COMT From *A. thaliana*

**FIGURE 2:**
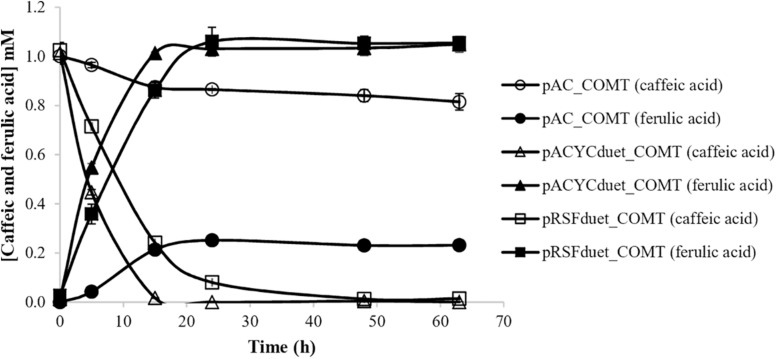
Production of ferulic acid from caffeic acid in *E. coli* BL21 (DE3) using different expression vectors. COMT, caffeic acid O-methyltransferase.

Different enzymes can be used to produce curcumin from tyrosine (Figure 1). In a previous study, 4CL was used to produce caffeoyl-CoA from caffeic acid and CCoAOMT was used to convert caffeoyl-CoA to feruloyl-CoA (Rodrigues et al., 2015b). Although the production of caffeic acid as intermediate in the curcuminoids pathway allowed to produce curcumin from tyrosine for the first time, the production obtained was residual. Since the initial steps of the biosynthetic pathway (from tyrosine to caffeic acid) and the last steps of the pathway (from ferulic acid to curcumin) were found to be very efficient, it was hypothesized that the combination of 4CL and CCoAOMT was the main bottleneck of the pathway. Therefore, in the newly designed pathway a 4CL was used to convert ferulic acid to feruloyl-CoA similarly to when curcumin was produced directly from this substrate. 4CL, more specifically 4CL1 from *A. thaliana*, although not reported to have higher affinity to ferulic acid than to caffeic acid (Ehlting et al., 1999), when combined with DCS and CURS1 was found to produce high concentrations of curcumin from ferulic acid (Rodrigues et al., 2015b; Couto et al., 2017). To further complete the pathway, it was important to find an efficient enzyme to convert caffeic acid to ferulic acid. COMT from *A. thaliana* was previously reported to be very efficient in that conversion step (Choi et al., 2011; Kang et al., 2012, 2015). Additionally, it has been reported to have more activity toward caffeic acid compared to other COMT enzymes recently characterized (Byeon et al., 2014; Dwivedi et al., 2014; Wiens and De Luca, 2016). Therefore, COMT gene from *A. thaliana* was herein codon-optimized and cloned into three different plasmids. Methionine was added to M9 minimal medium in order to increase the supply of S-adenosylmethionine, the methyl donor required in methyltransferase reactions (Heo et al., 2017). The production of ferulic acid from caffeic acid for each case is illustrated in Figure 2. In two cases, COMT was found to completely convert caffeic to ferulic acid (1 mM) and this conversion was faster using pACYCduet_COMT compared to pRSFduet_COMT. In the other case (pAC_COMT), the production of ferulic acid only reached 0.23 mM most probably due to the promoter used, i.e., the constitutive pLac, which is weaker than the pT7lac one. The expression of COMT enzyme was confirmed by SDS-PAGE (Supplementary Figure S1). As expected, the expression was higher when pRSFduet-1 was used followed by pACYCduet-1 and pAC.

### Production of Ferulic Acid From Tyrosine Using Different Combinations of Plasmids – Module 1 of the Biosynthetic Pathway

**FIGURE 3:**
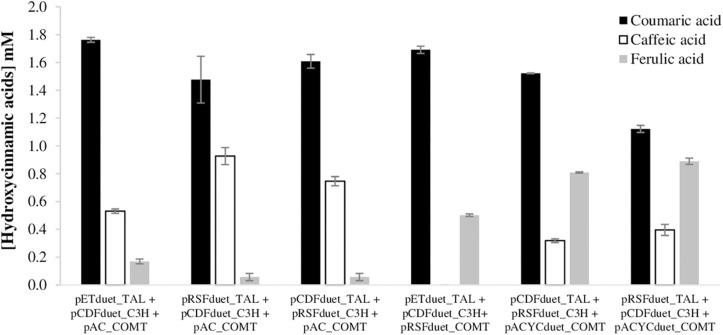
Production of the three hydroxycinnamic acids (coumaric, caffeic, and ferulic acids) in *E. coli* BL21 (DE3) using tyrosine as substrate, after 63 h of fermentation. TAL, tyrosine ammonia lyase; C3H, 4-coumarate 3-hydroxylase; COMT, caffeic acid O-methyltransferase.

After studying the ferulic acid production from caffeic acid, plasmids carrying the COMT gene were combined with previously constructed plasmids carrying TAL and C3H genes (Rodrigues et al., 2015a) to further evaluate the production of ferulic acid from tyrosine (Figure 3). As previously reported (Kang et al., 2012; Rodrigues et al., 2015a, b, 2017), coumaric acid accumulates in very high concentrations since the beginning of the fermentation while caffeic acid production is limited due to the C3H catalytic efficiency. Regarding ferulic acid productions, the plasmid combinations that included pAC_COMT led to very low concentrations of ferulic acid and consequently, a higher accumulation of caffeic acid in the culture medium was found. The combination of plasmids with pRSFduet_COMT led to a complete conversion of caffeic to ferulic acid. During the fermentation caffeic acid was never accumulated, it was automatically converted to ferulic acid. However, the highest ferulic acid titers were achieved when pACYCduet_COMT was used. In the combination that includes pRSFduet_COMT, the accumulation of coumaric acid was very high since the beginning of the fermentation (at 15 h was 1.95 mM), while in the cases where pACYCduet_COMT was present, the accumulation was not that high (at 15 h was 0.809–0.889 mM) (data not shown). This very high accumulation of the intermediate may be not beneficial to the production of ferulic acid since coumaric acid, and caffeic acid, are reported to be toxic to the cells at high concentrations (Watts et al., 2006; Furuya and Kino, 2013; Huang et al., 2013; Zhang and Stephanopoulos, 2013; Rodrigues et al., 2015a). Nevertheless, the highest ferulic acid concentration obtained from tyrosine was 889 μM (172.6 mg/L). This production is higher than the one reported by Wang et al. (2015) (28.8 mg/L) using TAL and C3H from *Saccharothrix espanaensis* and COMT from *Medicago sativa*. Other studies using a tyrosine overproducing *E. coli* strain carrying TAL and C3H from *S. espanaensis* and COMT from *A. thaliana* reported ferulic acid titers in the same range of this study (123–196 mg/L) (Kang et al., 2012, 2015). Indeed, the use of a tyrosine overproducing strain can be an advantageous strategy not only from an economic point of view, but also to improve ferulic acid production without accumulating high amounts of intermediates (coumaric and caffeic acid) as we observed in this study. The engineering of aromatic carboxylic acids efflux pumps involved in the excretion of hydroxycinnamic acids (such as *aaeXAB* operon) can also increase *E. coli* tolerance to hydroxycinnamic acids (Vargas-Tah and Gosset, 2015).

### Production of Curcumin From Ferulic Acid – Module 2 of the Biosynthetic Pathway

**FIGURE 4:**
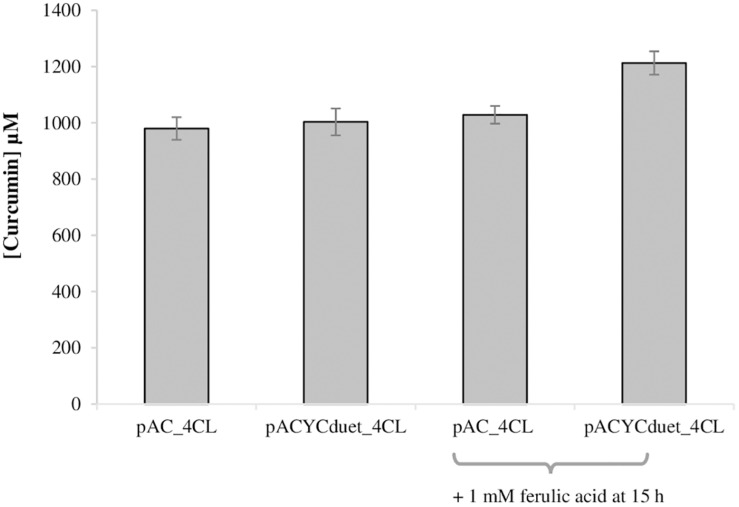
Production of curcumin from ferulic acid in *E. coli* BL21 (DE3) using different plasmids and conditions, after 63 h of fermentation. pRSFduet_CURS1 and pCDFduet_DCS were combined with pAC_4CL (1,3) or pACYCDuet_4CL (2,4). Ferulic acid was added at a concentration of 2 mM at time 0 of induction in M9 (1–4) and 1 mM of ferulic acid was also added after 15 h (3,4). 4CL, 4-coumarate-CoA ligase; DCS, diketide-CoA synthase; CURS1, curcumin synthase 1.

The second module of the pathway (from ferulic acid to curcumin) was also optimized. Previously, 4CL gene was expressed in pAC plasmid (Wang et al., 2013; Rodrigues et al., 2015b, 2017; Couto et al., 2017). However, since pACYCduet plasmid allowed to obtained better results in the production of ferulic acid than pAC (Figure 2), 4CL gene was also cloned in pACYCduet. Afterward, pACYCduet_4CL was combined with pCDFduet_DCS and pRSFduet_CURS1. As can be observed in Figure 4, the curcumin production when using pACYCduet_4CL instead of pAC_4CL improved but not significantly as the yield was already around 100%. Therefore, more ferulic acid (1 mM) was added at 15 h in both cases. The extra ferulic acid was not added in the beginning of the assay to avoid an unnecessary toxicity to the cells. A 20.9% increase of curcumin production corresponding to 1212.7 μM (446.7 mg/L), the highest concentration reported so far using heterologous production, was obtained. Although we increased the concentration of the starter substrate (ferulic acid), the cells were not able to completely convert it to curcumin. A possible reason for this is the low availability of the precursor malonyl-CoA (Xu et al., 2012, 2013; Machado et al., 2014; Lv et al., 2019) that is naturally synthesized in *E. coli* for the production of fatty acids and phospholipids and that is also needed for the production of curcuminoids (Figure 1). Therefore, in the future, to achieve high concentrations of curcumin from ferulic acid it will be important to also increase malonyl-CoA availability using well-established methods (Xu et al., 2012, 2013; Rodrigues et al., 2015c; Jones et al., 2017; Fang et al., 2018; Lv et al., 2019).

While curcumin (or other curcuminoids) production from ferulic acid (or other hydroxycinnamic acids) is not our main goal since this can be expensive at an industrial scale, it can be necessary for some applications given that obtaining very pure curcumin (or other curcuminoids) from plant extraction or chemical synthesis is extremely difficult and/or expensive (Friesen et al., 2019; Neven, 2019). Its heterologous production from glucose to obtain high purities is also not possible yet, mainly due to the involved enzymes (4CL, DCS, CURS1) broad specificity. On the contrary, the direct addition of ferulic acid (or other hydroxycinnamic acid) leads to the production of pure curcumin (or other curcuminoid). Ultimately, these pure compounds can be used, for example, to determine their therapeutic activity either alone or in different combinations.

### Curcuminoids Production by *E. coli* Mono-Culture System – Whole Biosynthetic Pathway

**FIGURE 5:**
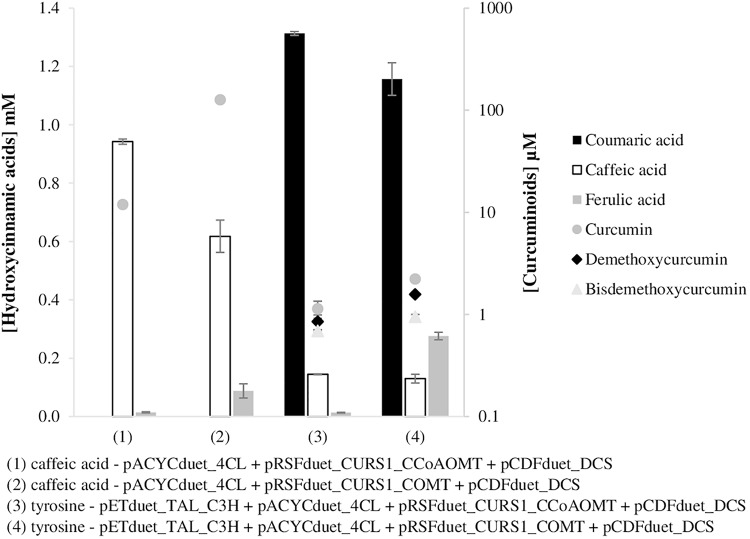
Curcuminoids production in *E. coli* BL21 (DE3) (mono-culture) using caffeic acid (1, 2) and tyrosine (3,4) as substrates, after 63 h of fermentation. Two different pathways were tested with two different enzymes, CCoAOMT (1,3) and COMT (2,4). TAL, tyrosine ammonia lyase; C3H, 4-coumarate 3-hydroxylase; COMT, caffeic acid O-methyltransferase; CCoAOMT, caffeoyl-CoA O-methyltransferase; 4CL, 4-coumarate-CoA ligase; DCS, diketide-CoA synthase; CURS1, curcumin synthase 1.

After optimizing both pathway modules, the production of curcuminoids from caffeic acid and tyrosine (Figure 5) was evaluated in a mono-culture system. For that purpose, the COMT gene was cloned in the same plasmid of CURS1 gene similarly to what was previously done for CCoAOMT (Rodrigues et al., 2015b) and the pETduet_TAL_C3H plasmid was also combined. pETduet-1 plasmid carrying the genes of interest (TAL and C3H) was chosen due to the compatible antibiotic resistance and replication origin. In addition, the pathway with pACYCduet_4CL was also tested with CCoAOMT. The curcuminoids production using CCoAOMT was improved comparing to the results previously reported (Rodrigues et al., 2015b) probably not only due to the cloning of 4CL gene in pACYCduet-1, but also to the use of a different host, *E. coli* BL21 (DE3) instead of *E. coli* K-12 (DE3). In a previous work we found that *E. coli* BL21 (DE3) was a better host to produce curcumin (Couto et al., 2017).

Comparing the results obtained when CCoAOMT was used [Figure 5, experiments (1) and (3)] with the ones with COMT [Figure 5, experiments (2) and (4)], it was found that COMT enzyme leads to a higher curcumin (and other curcuminoids) production. Curcumin production using COMT enzyme was 10.6 and 2.0 times higher than using CCoAOMT when caffeic acid and tyrosine, respectively, were supplied. The pathway including COMT instead of CCoAOMT possibly has an increased feruloyl-CoA availability.

As expected, when caffeic acid (1 mM) was supplemented to produce curcumin, its production (126.5 μM) decreased significantly [Figure 5, experiment (2)] compared to the production obtained when ferulic acid was supplemented directly to the medium [Figure 4, experiment (2) – 1003 μM]. As can be observed in Figure 5, ferulic acid was produced in low amounts. When tyrosine was used as substrate the curcumin production was even lower (2.2 μM–0.82 mg/L), indeed it decreased 57 times. Even though, it was higher than the one reported by Wang et al. (2015) (0.6 mg/L) using a similar pathway but with COMT from *M. sativa* and CUS from *O. sativa*. The total concentration of curcuminoids produced in these experiments (Figure 5) from tyrosine was approximately 4.7 μM (1.7 mg/L).

### Curcuminoids Production by *E. coli* Co-culture System – Combination of Module 1 and Module 2 of the Biosynthetic Pathway

**FIGURE 6:**
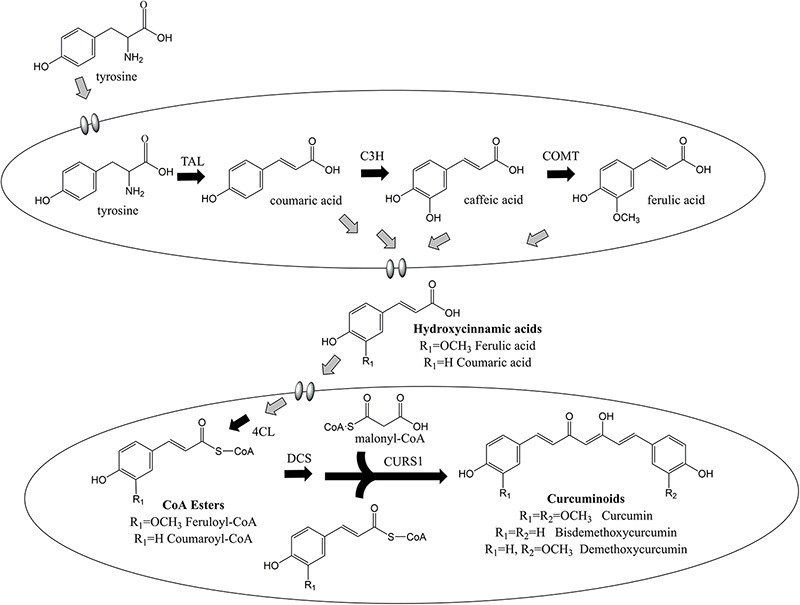
Cascade conversion of tyrosine to curcuminoids using a co-culture system. One *E. coli* strain converts tyrosine to ferulic acid and the other one converts ferulic acid and coumaric acid to different curcuminoids. TAL, tyrosine ammonia lyase; C3H, 4-coumarate 3-hydroxylase; COMT, caffeic acid O-methyltransferase; 4CL, 4-coumarate-CoA ligase; DCS, diketide-CoA synthase; CURS1, curcumin synthase.

**FIGURE 7:**
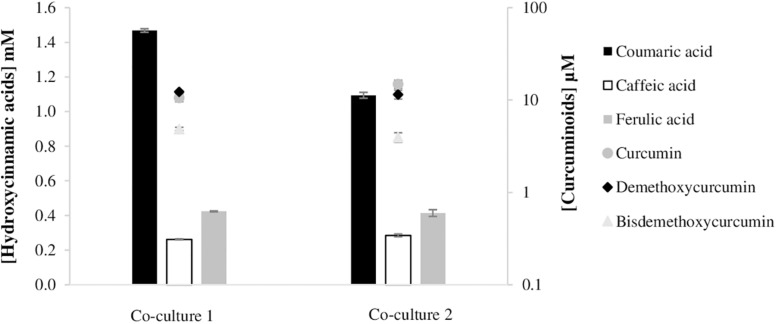
Curcuminoids production in co-culture experiments, after 63 h of fermentation. Each *E. coli* BL21 (DE3) carried a module of the pathway. The difference between the two co-cultures was the first module. Co-culture 1 contained one *E. coli* carrying pCDFduet_TAL, pRSFduet_C3H and pACYCduet_COMT, and co-culture 2 one *E. coli* carrying pRSFduet_TAL, pCDFduet_C3H and pACYCduet_COMT. The second module of the pathway was common to both co-cultures and was composed of pACYCduet_4CL, pCDFduet_DCS and pRSFduet_CURS1. TAL, tyrosine ammonia lyase; C3H, 4-coumarate 3-hydroxylase; COMT, caffeic acid O-methyltransferase; 4CL, 4-coumarate-CoA ligase; DCS, diketide-CoA synthase; CURS1, curcumin synthase 1.

The decrease observed in the curcuminoids production by *E. coli* mono-culture from tyrosine was predictable since, generally, as the complexity of the metabolic biosynthetic pathways increases, the metabolic load to the cell increases resulting in lower growth rates and productivity. However, the increase of pathway complexity and its further optimization is of upmost importance as feeding hydroxycinnamic acids to produce curcumin and/or curcuminoids is not economically feasible. Curcuminoids heterologous production will only reach industrial scale in the future if simple carbon sources, such as glucose, can be used as substrate by a tyrosine overproducing *E. coli* strain and if high productions can be obtained. Therefore, in order to distribute the metabolic burden and increase the production efficiency, a co-culture strategy was designed. For this purpose, two *E. coli* BL21 (DE3) strains were used, one carrying the pathway to convert tyrosine to ferulic acid, and the other carrying the second module of the pathway to convert ferulic acid (and/or coumaric acid) to curcuminoids (Figure 6). For the first module of the pathway, two strains that showed previously (Figure 3) high production of ferulic acid were used – the strain carrying pCDFduet_TAL, pRSFduet_C3H and pACYCduet_COMT and the strain carrying pRSFduet_TAL, pCDFduet_C3H and pACYCduet_COMT (Figure 7). These strains were combined with one strain that carried pACYCduet_4CL, pCDFduet_DCS and pRSFduet_CURS1 and these combinations are from now on designated co-culture 1 and co-culture 2, respectively. Other strains carrying other combinations of the first module of the pathway (Figure 3) were not chosen for further experiments since it was considered that the concentration of ferulic acid that they produced was very low and therefore, not enough to have significant titers of curcuminoids. Moreover, the strain carrying pETduet_TAL, pCDFduet_C3H and pRSFduet_COMT (Figure 3) was not chosen for further experiments since it was not able to grow when combined with the strain that carried the second module of the pathway due to selection mark incompatibility.

The division of labor between the two strains allowed to improve the production of curcuminoids. The highest curcumin production with these co-culture strategies was 14.9 μM and this was 6.8 times higher than the one achieved using the mono-culture system [Figure 5, experiment (4)]. In addition, the total curcuminoids production also increased 6.4 times. This type of improvement was also observed by Fang et al. (2018) that used co-culture engineering to produce bisdemethoxycurcumin.

### Curcuminoids Production by Mono-Culture and Co-culture Systems After Reducing Metabolic Burden Caused by Multiple Plasmids

**FIGURE 8:**
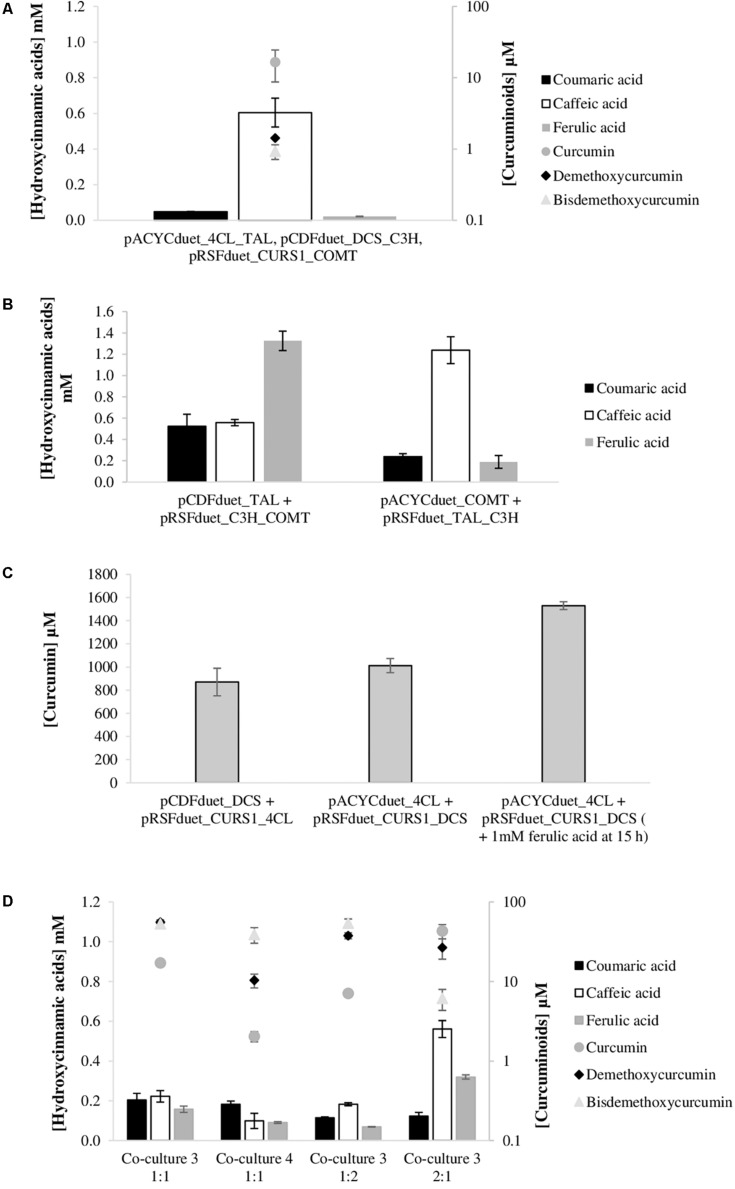
Curcuminoids and hydroxycinnamic acids production by *E. coli* at 63 h after reducing the number of plasmids used. **(A)** Production of curcuminoids by *E. coli* BL21 (DE3) mono-culture system from tyrosine and carrying pACYCduet_4CL_TAL, pCDFduet_DCS_C3H and pRSFduet_CURS1_COMT. **(B)** Production of hydroxycinnamic acids by *E. coli* BL21 (DE3) from tyrosine and carrying pCDFduet_TAL and pRSFduet_C3H_COMT or pRSFduet_TAL_C3H and pACYCduet_COMT. **(C)** Production of curcumin by *E. coli* BL21 (DE3) Δ*lacZ* from ferulic acid and carrying pCDFduet_DCS and pRSFduet_CURS1_4CL or pACYCduet_4CL and pRSFduet_CURS1_DCS. **(D)** Production of curcuminoids by co-culture system from tyrosine. In co-culture 3, *E. coli* BL21 (DE3) carried pCDFuet_TAL and pRSFduet_C3H_COMT and *E. coli* BL21 (DE3) Δ*lacZ* carried pCDFduet_DCS and pRSFduet_CURS1_4CL. In co-culture 4, *E. coli* BL21 (DE3) carried pRSFduet_TAL_C3H and pACYCduet_COMT and *E. coli* BL21 (DE3) Δ*lacZ* carried pACYCduet_4CL and pRSFduet_CURS1_DCS. Co-culture 3 was tested using different inoculation ratios (1:1, 2:1 and 1:2). TAL, tyrosine ammonia lyase; C3H, 4-coumarate 3-hydroxylase; COMT, caffeic acid O-methyltransferase; 4CL, 4-coumarate-CoA ligase; DCS, diketide-CoA synthase; CURS1, curcumin synthase 1.

Plasmid replication and maintenance imposes a metabolic burden on the host cells since it takes over cell resources such as carbon building blocks and energy molecules (adenosine triphosphate, nicotinamide adenine dinucleotide, nicotinamide adenine dinucleotide phosphate, among others). Reducing the number of plasmids reduces the load to the cell and can increase the productivity of the engineered strain. Since the plasmids used in this study are Duet plasmids (have two multiple cloning sites with two T7 promoters) (Novagen), another type of experimental design could be evaluated. The mono-culture system could be improved by cloning for instance TAL and C3H genes in the plasmids containing 4CL and DCS genes, reducing the number of plasmids used and, consequently, the metabolic burden. Having this goal in mind, the necessary cloning and experiments were performed. As can be observed in Figure 8A, that gathers the mono-culture system results after reducing the number of plasmids from four to three, the curcuminoids production improved significantly. Using this strategy, the curcumin production was 7.5 times higher than the one obtained using four plasmids [Figure 5, experiment (4)]. This production (16.6 μM; 6.1 mg/L) was the highest production obtained so far from tyrosine using mono-culture system. The highest production of curcumin from tyrosine reported until now was 0.6 mg/L by Wang et al. (2015). From Figure 8A, it is possible to verify that the production of the other curcuminoids was very similar to the one previously achieved [Figure 5, experiment (4)]. The other curcuminoids (bisdemethoxycurcumin and curcumin) need coumaric acid accumulation to be further produced. However, according to the results obtained, coumaric acid was very efficiently converted to caffeic acid. Indeed, it was never significantly accumulated during the 63 h (data not shown).

Co-culture systems might also be improved by decreasing the number of plasmids used. Each strain may carry only two plasmids with, for example, three genes reducing even more the metabolic burden. From Figures 8B,C, it is possible to observe the results achieved for each module of the pathway. Two combinations of plasmids were tested for each module. For the production of ferulic acid from tyrosine, *E. coli* carrying pCDFduet_TAL and pRSFduet_C3H_COMT was the one that allowed to obtain higher productions of ferulic acid (1325.1 μM). When compared to the strategy that produced more ferulic acid using three plasmids (pRSFduet_TAL + pCDFDuet_C3H + pACYCduet_COMT) (Figure 3), the production increased 49%, which is very significant. This is by far the highest ferulic acid titer obtained using tyrosine as substrate. This improvement is of interest for all of those working in the production not only of curcuminoids but other relevant compounds that are produced from ferulic acid such as coumarins, lignans, vanillin, among others. Using the other combination of plasmids, ferulic acid production was very low while caffeic acid reached a very high concentration (1238.2 μM). This concentration is also higher than the ones previously reported using C3H gene (Zhang and Stephanopoulos, 2013; Rodrigues et al., 2015a).

As previously mentioned, the design of the second module of the pathway was also rethought (Figure 8C). To use only two plasmids, 4CL and DCS genes were cloned in pRSFduet_CURS1 and two combinations of plasmids were tested, namely pCDFduet_DCS + pRSFduet_CURS1_4CL and pACYCduet_4CL + pRSFduet_CURS1_DCS. These combinations allowed to achieve very high concentrations and yields of curcumin as when three plasmids were used (Figure 4). However, when more substrate was added after 15 h, the conversion of curcumin was significantly higher than the one obtained previously with three plasmids. This reduction in the metabolic burden allowed to obtain the highest curcumin concentration reported to date, 1529.5 μM (563.4 mg/L), with a percent yield of 100%. This shows that, at these levels of curcumin production, contrary to what was hypothesized, the precursor malonyl-CoA is not limitant.

After testing both modules of the pathway separately, the complete pathway was tested using a co-culture system. In this case, each *E. coli* carried a module of the pathway but with only two plasmids instead of three as was presented in Figure 7. Using the plasmids presented in Figures 8B,C, only two combinations (named co-culture 3 and co-culture 4) could be tested since for both strains to grow together they needed the same antibiotic selection. Co-culture 3 contained pCDFduet_TAL and pRSFduet_C3H_COMT in one *E. coli* and pCDFduet_DCS and pRSFduet_CURS1_4CL in the other. Co-culture 4 was composed by one *E. coli* carrying pRSFduet_TAL_C3H and pACYCduet_COMT and another one carrying pACYCduet_4CL and pRSFduet_CURS1_DCS. These co-cultures allowed to produce higher concentrations of total curcuminoids than the ones previously tested (Figure 7). Co-culture 3 was the one that allowed to obtain higher production (125.8 μM). This production was 6.6 and 4.2 times higher than the ones obtained with the optimized mono-culture (Figure 8A) and with the other co-cultures tested (Figure 7), respectively. However, the curcumin production (17.1 μM) did not increase significantly. Coumaric acid was highly accumulated in the beginning of the fermentation (Supplementary Figure S2) leading to high productions of bisdemethoxycurcumin, that is produced using two molecules of coumaric acid. In co-culture 3 ferulic acid was not accumulated in high amounts during fermentation and, consequently, curcumin was not significantly produced. This was even more evident for co-culture 4 that produced ferulic acid in even lower amounts leading to only very high titers of demethoxycurcumin. It is very important that the intermediates are highly accumulated since the division of labor between the two strains may disrupt natural channels and dilute the intermediate concentrations inside the cell that uses it as substrate (Roell et al., 2019).

In these fermentations, the second module of the pathway was inserted in a *E. coli* BL21 (DE3) strain with the *lacZ* gene disrupted. This disruption does not allow the production of β-galactosidase that converts X-Gal into a blue insoluble pigment. Therefore, it is possible to distinguish both strains when X-Gal is present – the strain carrying the second module forms white colonies while the other one forms blue colonies (Zhang et al., 2015; Li et al., 2019). By plating fermentation samples, it was possible to estimate the strain-to-strain ratio in the co-cultures. In parallel, the samples were also plated in medium with ferulic acid. This also allowed to distinguish the strains through differences in color (orange-yellow/white), but the colonies were more difficult to distinguish, depending on the amount of curcumin produced by each colony. Anyway, the results obtained through the two methods were very consistent. Although the strains were inoculated in the same proportion, it was observed that after inducing the protein expression in LB with IPTG the population started to change and after 5 h of induction the ratio was 1.3%:98.7% (strain carrying first module: strain carrying second module). This was not expected since both strains when inoculated separately exhibited approximately the same growth rate (≈0.8 h^–1^). However, this type of change in the ratio between the co-culture strains was also previously observed by Li et al. (2019) and is considered normal since both strains are competing for the same carbon source. The balanced growth of the co-cultures strains is very hard to maintain (Zhang and Wang, 2016). From the results it is possible to conclude that the strain carrying the first module of the pathway was at growth disadvantage. After 24 h in M9 the population started to change and at 63 h the proportion was around 20%: 80%. This change may be justified by the accumulation of curcuminoids inside the strains carrying the second module of the pathway. The accumulation of curcuminoids may decrease the cells viability due to their known antibacterial activity. In the future, the engineering of transporter proteins to excrete curcuminoids to the extracellular medium can be very valuable. Zhao et al. (2018) overexpressed 10 transport genes (multidrug efflux pump proteins, transporters and outer membrane proteins) and almost doubled resveratrol production. A similar approach can improve curcuminoids yield by decreasing their toxicity to the cells.

The population behavior in co-culture motivated the study of different inoculation ratios (1:2 and 2:1) of co-culture 3, the one that allowed higher production of curcuminoids (Figure 8D). When the strain containing the first module was inoculated at a lower ratio (1:2), the production of hydroxycinnamic acids was very low during the fermentation and the conversion of coumaric acid to caffeic acid and ferulic acid was not very efficient. Therefore, bisdemethoxycurcumin and demethoxycurcumin were the curcuminoids produced in higher amounts. The total curcuminoids concentration (98.9 μM) and proportion was not very different from the one obtained for the ratio 1:1. However, when the strain containing the first module of the pathway was present in a higher percentage in the beginning of the fermentation (ratio 2:1) the proportion of curcuminoids obtained was different. Curcumin (43.2 μM) was the most produced curcuminoid followed by demethoxycurcumin. Bisdemethoxycurcumin, that depends exclusively on the coumaric acid availability was produced in lower amounts because during fermentation coumaric acid was efficiently converted to ferulic acid (Supplementary Figure S2). Although this ratio (2:1) did not allow to increase the number of total curcuminoids produced (76.0 μM), by increasing the amount of hydroxycinnamic acids produced during fermentation it allowed to obtain 2.5-fold more curcumin. The amount produced here was the highest amount of curcumin obtained from tyrosine to date. The strain-to-strain ratio was also estimated for both ratios tested (1:2 and 2:1) and it was verified, for both cases, that at time 0 in M9 the ratio was 33%: 66% which shows that the strain carrying the first module of the pathway is clearly in disadvantage and even when it is inoculated in higher proportion the strain carrying the second module of the pathway prevailed. Nevertheless, the higher proportion at inoculation time allowed to produce the proteins needed to obtained higher production of hydroxycinnamic acids, including ferulic acid that was converted to curcumin.

The titers herein presented represent 2550 and 6817% improvements for curcumin and total curcuminoids, respectively, over the highest titers reported in the literature to date using an equivalent biosynthetic pathway (Wang et al., 2015). The productions are also significantly higher than the ones achieved by Kang et al. (2018), but they used a tyrosine overproducing strain whereby they are not comparable. In the future other inoculation ratios can be tested to improve the productions and/or to favor the production of one curcuminoid in relation to the others. The use of a co-culture system only in the M9 phase of fermentation can also be advantageous. This way the strains will not need to compete in LB when they are expressing the heterologous proteins and high amounts of proteins from both modules will be present in the beginning of the fermentation in M9. In addition, the use of statistical models to identify optimal expression patterns can be very useful to in the future improve metabolic pathway efficiency (Xu et al., 2017).

## Conclusion

In this work, we successfully designed, constructed and optimized a heterologous pathway to produce curcuminoids in *E. coli*. This biosynthetic pathway includes 6 different enzymes that catalyze in some cases more than one reaction. One of these enzymes was COMT that converts caffeic acid to ferulic acid. We optimized the ferulic acid production from tyrosine using COMT and 1325.1 μM (257.3 mg/L) of ferulic acid were produced. In addition, we obtained the highest curcumin production ever reported in *E. coli* when ferulic acid is used as substrate. Around 1529.5 μM (563.4 mg/L) of curcumin was produced corresponding to a 59.4% increase compared to previously reported productions. Furthermore, the curcuminoids production from tyrosine using COMT was compared to its production using CCoAOMT (which converts caffeoyl-CoA to feruloyl-CoA) reported in previous studies. The production of curcuminoids using COMT was significantly higher than the one using CCoAOMT in the biosynthetic pathway. Using COMT enzyme in a monoculture strategy, the production of curcumin increased to 16.6 μM (6.1 mg/L). Finally, we also used a co-culture strategy to produce curcuminoids. One *E. coli* strain was used to produce ferulic acid from tyrosine, while the other strain converted the ferulic acid (and/or coumaric acid) produced and released to the medium to produce curcumin (and/or other curcuminoids). This strategy allowed to increase the amount of curcumin (43.2 μM; 15.9 mg/L) and total curcuminoids (125.8 μM; 41.5 mg/L) produced, possibly due to a decreased metabolic burden to each cell. The productions were very dependent on the inoculation ratios of the two cultures. To our knowledge, the productions herein reported are the highest productions of curcumin/curcuminoids obtained until now. These results are of interest for all of those working not only with the heterologous production of curcuminoids, but also other more complex polyphenolic compounds or plant secondary metabolites.

Although the results obtained are very promising, further improvements can still be made. In the future, this artificial biosynthetic pathway should be tested using a tyrosine overproducing *E. coli* strain to carry the first module. This strain will be more suitable for an industrial scale since phenylpropanoic acid substrates are expensive and have low solubility in aqueous solutions. Moreover, using this strain would allow to reduce the possible hydroxycinnamic acids toxicity effects as they will be accumulated in a lesser extent. Future metabolic optimizations should also include some step(s) to increase the malonyl-CoA availability that is generally low for recombinant pathways. The engineering of transporter proteins to decrease diffusion limitations and increase *E. coli* tolerance to hydroxycinnamic acids and curcuminoids leading to a higher production of this compounds is also of utmost importance. Finally, to produce pure curcumin from tyrosine or glucose, less promiscuous enzymes need to be found or the existing ones need to be further engineered.

## Data Availability Statement

All datasets generated for this study are included in the article/Supplementary Material.

## Author Contributions

JR performed most of the experiments and data analysis, and conceived, designed, and drafted the manuscript. DG performed some of the experiments and data analysis. LR coordinated the study and team, and provided feedback and suggestions on the manuscript. All authors gave final approval for publication.

## Conflict of Interest

The authors declare that the research was conducted in the absence of any commercial or financial relationships that could be construed as a potential conflict of interest.
