# Supplementary material for: A Combinatorial Approach to Optimize the Production of Curcuminoids From Tyrosine in Escherichia coli
Source: Front Bioeng Biotechnol. 2020 Feb 7;8:59. doi: 10.3389/fbioe.2020.00059 (PMC7019186; doi:10.3389/fbioe.2020.00059)
Supplement: Supplementary file 1 [file Data_Sheet_1.pdf]

Supplementary Material

Table S1. Gene sequence of COMT with codon optimization.

Gene	Sequence
COMT<br>(*Arabidopsis thaliana*)	ATGGGCTCGACCGCCGAAACGCAGCTGACGCCAGTGCAGGTTACCGATGACGAAGCGGC<br>ACTGTTTGCCATGCAGCTGGCGTCCGCCTCGGTCTTGCCAATGGCGCTCAAAAGCGCGTT<br>GGAGCTGGATCTGCTCGAAATTATGGCGAAAAATGGCTCGCCGATGTCCCCGACCGAGA<br>TCGCCAGTAAATTACCAACGAAAAATCCGGAGGCGCCGGTGATGCTCGACCGTATTCTG<br>CGTCTTTTGACCTCGTATAGTGTTTTAACCTGCAGTAATCGTAAATTATCGGGCGACGGT<br>GTGGAACGCATCTACGGTCTGGGCCCAGTTTGCAAATATCTGACGAAAAATGAGGACGG<br>TGTTAGCATCGCGGCCCTGTGTCTGATGAACCAGGATAAAGTTCTGATGGAATCCTGGTA<br>TCACCTCAAAGATGCGATCCTGGATGGCGGCATTCCGTTTAACAAAGCCTATGGTATGAG<br>CGCCTTCGAATATCATGGTACTGACCCACGTTTTAATAAAGTGTTTAACAATGGCATGAG<br>TAATCATTCGACCATTACCATGAAAAAGATTTTAGAGACTTACAAGGGCTTTGAAGGCTT<br>AACCTCCCTGGTTGATGTGGGCGGCGGCATCGGCGCGACCCTGAAAATGATTGTGAGCA<br>AATATCCGAATCTGAAAGGTATTAACTTTGATCTTCCGCATGTTATCGAGGACGCACCGA<br>GTCACCCAGGTATCGAGCATGTGGGCGGCGACATGTTTGTGAGTGTTCCGAAGGGCGAC<br>GCGATCTTTATGAAATGGATTTGTCATGATTGGAGTGATGAGCATTGTGTCAAATTTCTT<br>AAAAATTGTTATGAATCGTTACCAGAGGATGGTAAAGTTATTCTGGCAGAATGCATTTTA<br>CCAGAAACCCCGGATTCCAGCTTGTCCACCAAACAGGTCGTGCACGTTGACTGTATCATG<br>TTAGCCCATAACCCAGGCGGCAAAGAGCGCACCGAAAAAGAATTCGAGGCGTTGGCGA<br>AAGCAAGCGGCTTCAAAGGTATCAAGGTTGTCTGTGATGCCTTTGGTGTGAATCTGATTG<br>AACTCCTGAAGAAACTGTAA

Table S2. Primers for PCR amplification of the genes of the biosynthetic pathway (forward (FW) and reverse (REV) primers) and sequencing.

Primer name	Primer sequence†	Restriction enzyme site
COMT_pAC_FW	AAAAA*TCTAGA***ATG**GGCTCGACCGCCG	XbaI
COMT_pAC_REV	AAAAA*GCGGCCGC***TTA**CAGTTTCTTCAGGAGTTCAATCAGA	NotI
COMT_duet_FW	AAAAA*GGATCC*A**ATG**GGCTCGACCGCCG	BamHI
COMT_duet_REV	AAAAA*CTCGAG***TTA**CAGTTTCTTCAGGAGTTCAATCAGA	XhoI
COMT_duet_FW2	AAAAA*CAT**ATG***GGCTCGACCGCCG	NdeI
4CL_FW	AAAAA*GGATCC*G**ATG**GCGCCACAAGAACAAG	BamHI
4CL_REV	AAAAA*AAGCTT***TCA**CAATCCATTTGCTAGTTTTG	HindIII
4CL_FW2	AAAAA*CATATG***ATG**GCGCCACAAGAACA	NdeI
4CL_REV2	AAAAA*CTCGAG***TCA**CAATCCATTTGCTAGTTTTGC	XhoI
TAL_FW	TTTTT*GATATC*C**ATG**GCTCCGCGTCCG	EcoRV
TAL_REV	TTTTT*CTCGAG***TTA**TGCCAGCATTTTCA	XhoI
C3H_FW	AAAAA*CAT**ATG***ACGATTACCTCTCCGG	NdeI
C3H_REV	TTTTTT*CTCGAG*CGTGCCCGGGTTAATCAG	XhoI
DCS_FW	TTTTT*CATATG***ATG**GAAGCTAACGGTTACCG	NdeI
DCS_REV	AAAAA*CTCGAG***TTA**GTTCAGACGGCAAGAGTG	XhoI
pAC_seq_FW	ACTATCGACTACGCGATCAT	-
pAC_seq_REV	ATCGGTGATGTCGGCGATA	-
pDuet_seq_FW	GGATCTCGACGCTCTCCCTT	-
pDuet_seq_FW2	GTACACGGCCGCATAATCG	-
pDuet_seq_REV	CTAGTTATTGCTCAGCGGT	-

† Start and stop codons in **bold**; restriction sites in *italic*; In order for the sequence to remain in frame one base was occasionally added between the restriction site and the gene start codon.

Table S3. Primers (forward (FW) and reverse (REV)) used in the construction and verification of pKDsgRNA-*lacZ*.

**Primer name**	**Primer sequence†**
Fragment1_Protospacer_FW	AGATGTGCGGCGAGTTGCGTGACTACCTAC**gttttagagctagaaatagcaag**
Fragment1_REV*	**tttataacctccttagagctcga**
Fragment2_FW*	**ccaattgtccatattgcatca**
Fragment2_Protospacer_REV	GTAGGTAGTCACGCAACTCGCCGCACATCT**gtgctcagtatctctatcactga**
pKD_Seq5*	CAGTGAATGGGGGTAAATGG
sgRNA_R*	GCCTGCAGTCTAGACTCGAG

* primers described in Reisch and Prather (2015)

† The underlined sequences (30 nt) represent the protospacer described in Chung et al. (2017); sequences in **bold** are complementary to plasmid pKDsg-p15A

Table S4. Oligonucleotide used for the disruption of *lacZ* gene. The nucleotides that are homologous to the upstream region of *lacZ* and to its the intergenic region are underlined and **bold**, respectively.

**Oligonucleotide**	**Sequence**
**Oligo_*lacZ***	TCGTATGTTGTGTGGAATTGTGAGCGGATAACAATTTCACACAGGAAACA**CGCGTGGATGAAGACCAGCCCTTCCCGGCTGTGCCGAAATGGTCCATCAA**

Table S5. Primers (forward (FW) and reverse (REV)) used to verify *lacZ* disruption, described in Chung et al. (2017).

**Primer name**	**Primer sequence**
LacZ_FW	GTTGGCCGATTCATTAATGCA
LacZ_REV	TACTGTGAGCCAGAGTTGCCC

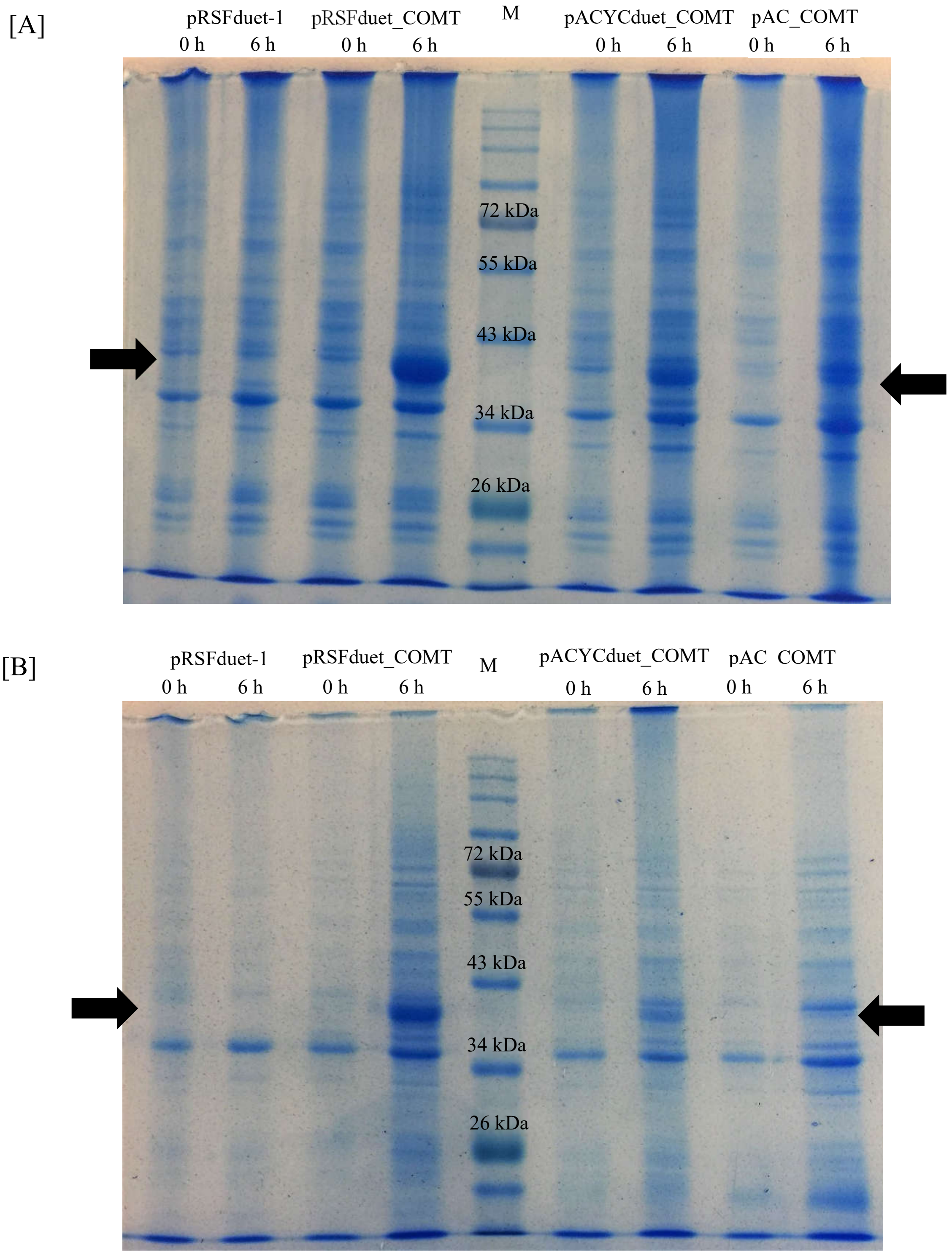

Figure S1: Protein SDS gels showing caffeic acid O-methyl transferase (COMT) expression using different plasmids (pRSFduet-1, pACYCduet-1 and pAC) at time zero of induction and after 6 h of induction. COMT is expected around 39.62. Arrows indicate where it is possible to observe the bands of interest. M: marker (Color protein standard broad range – NEB). Soluble (A) and insoluble (B) fractions.

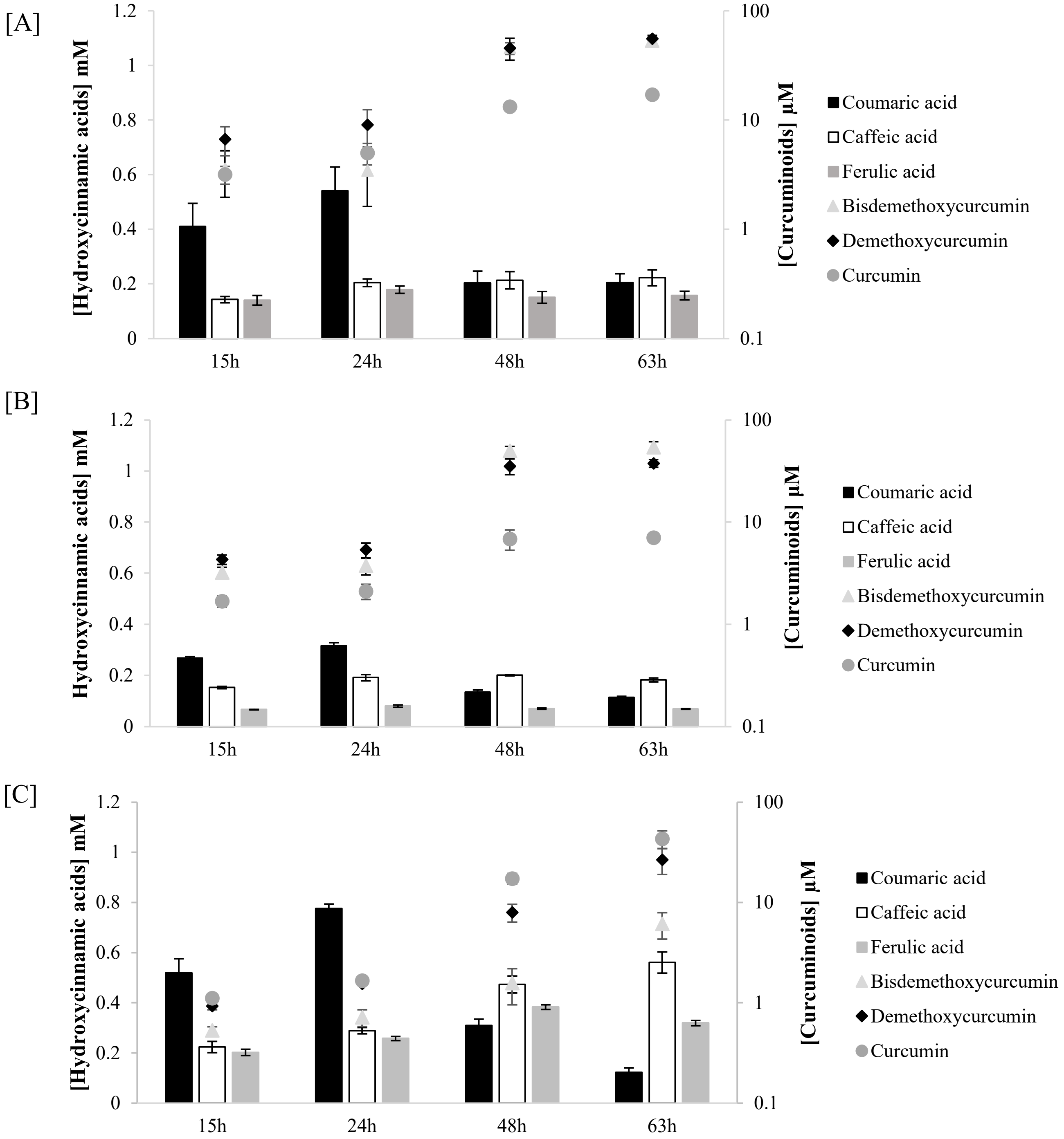

Figure S2. Curcuminoids and hydroxycinnamic acids production by co-culture 3 (*E. coli* BL21 (DE3) carried pCDFuet_TAL and pRSFduet_C3H_COMT and *E. coli* BL21 (DE3) *ΔlacZ* carried pCDFduet_DCS and pRSFduet_CURS1_4CL). Co-culture 3 was tested using different inoculation ratios: 1:1 (A), 1:2 (B) and 2:1 (C). TAL: tyrosine ammonia lyase, C3H: 4-coumarate 3-hydroxylase, COMT: caffeic acid O-methyltransferase, 4CL: 4-coumarate-CoA ligase, DCS: diketide-CoA synthase, CURS1: curcumin synthase 1.

Chung, M.E., Yeh, I., Sung, L.Y., Wu, M.Y., Chao, Y.P., Ng, I.S., et al. (2017). Enhanced integration of large DNA into *E. coli* chromosome by CRISPR/Cas9. *Biotechnol. Bioeng.* 114**,** 172-183. doi: 10.1002/bit.26056.

Reisch, C.R., and Prather, K.L. (2015). The no-SCAR (Scarless Cas9 Assisted Recombineering) system for genome editing in *Escherichia coli*. *Sci. Rep.* 5**,** 1-12. doi: 10.1038/srep15096.
